# What protein kinases are crucial for acantholysis and blister formation in pemphigus vulgaris? A systematic review

**DOI:** 10.1002/jcp.30784

**Published:** 2022-05-26

**Authors:** Adriano Brescacin, Zunaira Baig, Jaspreet Bhinder, Sen Lin, Lovejot Brar, Nicola Cirillo

**Affiliations:** ^1^ Melbourne Dental School, Faculty of Medicine, Dentistry & Health Sciences University of Melbourne Carlton Victoria Australia

**Keywords:** kinase inhibitors, pemphigus vulgaris, protein kinase, acantholysis

## Abstract

Pemphigus vulgaris (PV) is a potentially fatal autoimmune blistering disease characterized by cell–cell detachment (or acantholysis) and blister formation. While the signaling mechanisms that associate with skin/mucosal blistering are being elucidated, specific treatment strategies targeting PV‐specific pathomechanisms, particularly kinase signaling, have yet to be established. Hence, the aim of this review was to systematically evaluate molecules in the class of kinases that are essential for acantholysis and blister formation and are therefore candidates for targeted therapy. English articles from PubMed and Scopus databases were searched, and included in vitro, in vivo, and human studies that investigated the role of kinases in PV. We selected studies, extracted data and assessed risk of bias in duplicates and the results were reported according to the methodology outlined by the Preferred Reporting Items for Systematic Reviews and Meta‐analyses (PRISMA). The risk of bias assessment was performed on in vivo studies utilizing SYRCLE's risk of bias tool. Thirty‐five studies were included that satisfied the pathogenicity criterion of kinases in PV, the vast majority being experimental models that used PV sera (*n* = 13) and PV‐IgG (*n* = 22). Inhibition of kinase activity (p38MAPK, PKC, TK, c‐Src, EGFR, ERK, mTOR, BTK, and CDK2) was achieved mostly by pharmacological means. Overall, we found substantial evidence that kinase inhibition reduced PV‐associated phosphorylation events and keratinocyte disassociation, prevented acantholysis, and blocked blister formation. However, the scarce adherence to standardized reporting systems and the experimental protocols/models used did limit the internal and external validity of these studies. In summary, this systematic review highlighted the pathogenic intracellular events mediated by kinases in PV acantholysis and presented kinase signaling as a promising avenue for translational research. In particular, the molecules identified and discussed in this study represent potential candidates for the development of mechanism‐based interventions in PV.

## Graphical abstract

Branches of the kinome are involved in the pathogenesis of pemphigus vulgaris.

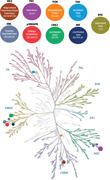

**Abbreviations**
BTK: Bruton tyrosine kinase
CDK: cyclin‐dependent kinase
ERK: extracellular signal‐regulated kinase
FAK: focal adhesion kinase
JNK: c‐Jun‐N‐terminal kinase
MK2: mitogen‐activated protein kinase‐activated protein kinase 2
P38 MAPK: p38 mitogen‐activated protein kinase
PI3K: phosphatidylinositol 3‐kinase 
PKC, protein kinase C; PLC: phospholipase C
TK: tyrosine kinase

## 1 INTRODUCTION

Pemphigus vulgaris (PV) is a chronic debilitating autoimmune disease that manifests as intraepithelial blistering and non‐healing erosions on both skin and mucosal membranes (Sanders, 2017). The incidence of PV varies based on geographical location, ethnicity, and gender. Its presentation ranges from ~1 to 3.5 cases per 100,000 individuals per year worldwide (Mao & Payne, 2008); more commonly impacting the 50‐ to 60‐year‐old cohort, females and individuals of Mediterranean Origin or Ashkenazi Jewish descent (Ruocco et al., 2013; Vodo et al., 2018). The incidence rate has been found to be increasing in the United Kingdom and Brazil (Porro et al., 2019). Historically, the disease was inevitably fatal before the emergence of systemic corticosteroids lowering the mortality rate to 10% (Bystryn & Rudolph, 2005). This form of nonspecific therapy remains the first line of treatment; however, this presents a significant medical concern as it is responsible for the mortality rate as a result of treatment complications (Cholera & Chainani‐Wu, 2016). Indeed, this perpetuates the need for a more specific therapy that improves treatment prognosis and results in lesser complications. Recently, there has been a shift from conventional therapies toward the development of more targeted therapies, and this cannot be achieved without a better understanding of the specific pathogenic mechanisms that are responsible for the disease.

The PV disease process was once believed to be primarily linked to autoantibodies disrupting the desmoglein 3 (Dsg3) and desmoglein 1 (Dsg1) cadherin family found on desmosomes, causing subsequent keratinocyte detachment, histologically known as acantholysis (Cirillo et al., 2012; Mavropoulos et al., 2013). However, it is now recognized that acantholysis is more complex than previously thought and our complete understanding of the molecular mechanism(s) remains unresolved (Berkowitz et al., 2006; Cirillo et al., 2012). Acantholysis has been proposed to be instigated by signaling events and phosphorylation of a number of target proteins found in keratinocytes. The binding of the pemphigus vulgaris immunoglobulin G (PV‐IgG) to a variety of self‐antigens, including desmosomal proteins, acetylcholine receptors (AChRs), and mitockondrial antigens activates phosphatidylcholine specific phospholipase C (PLC), which in turn elevates intracellular free calcium, and activates various kinases including epidermal growth factor receptor kinase (EFGRK), Src, protein kinase C (PKC), and p38 MAP Kinase (Cirillo et al., 2012). This triggers downstream activation of effector molecules that eventuate in the remodeling of cytoskeletal actin and intermediate filaments, apoptotic signaling, basal cell shrinkage, and transcriptional downregulation of desmosomal adhesion molecules (Cipolla et al., 2017; Cirillo et al., 2012).

Kinases are enzymes that orchestrate many functions involved in metabolism, cell cycle regulation, and differentiation. The phosphorylation of effector proteins by kinases is tightly regulated and any perturbation to this regulation may lead to a diseased state as is the case with PV (Ardito et al., 2017). Kinases are integral to the acantholytic mechanism of PV, thus their activation has been an area of interest with multiple large studies being performed in recent times. Despite the promising implications of key kinase pathways in a plethora of in‐vivo and in‐vitro studies, there has been limited translation to clinical human trials for kinase inhibitors (Murrell et al., 2021). The aim of this systematic review is to examine kinases that are pathogenically implicated in PV with a view of identifying potential evidence‐based therapeutic targets for PV.

## 2 METHODS

### 2.1 Search strategy

**Figure 1:**
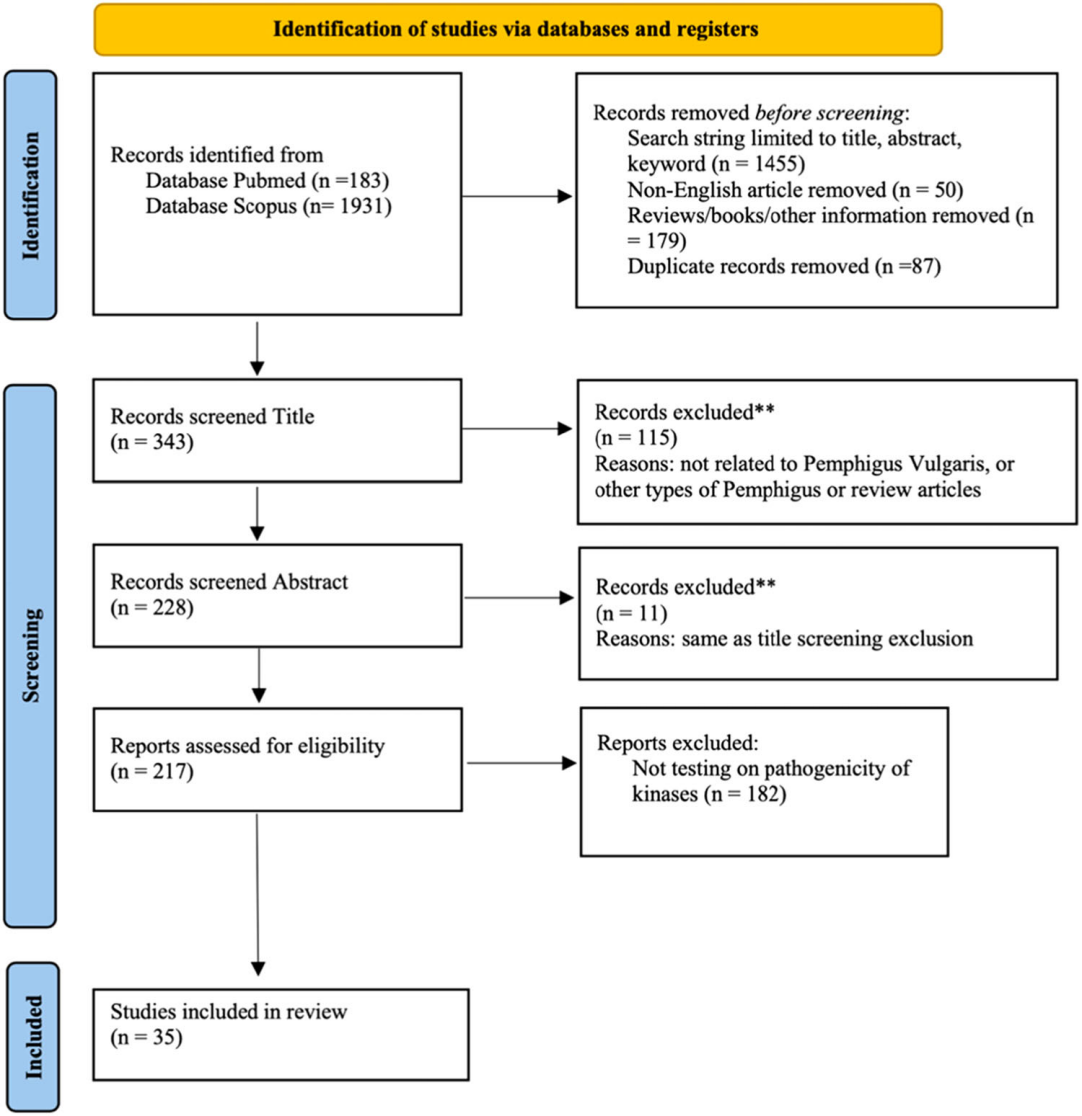
PRISMA 2020 flow diagram for new systematic reviews which included searches of databases, registers, and other sources.

This review follows the Preferred Reporting Items for Systematic Review and Meta‐Analyses (PRISMA) protocols (Page et al., 2021; Figure 1). It was designed to analyze evidence gathered in all experimental settings (in vitro, in vivo, and human studies) and not to directly evaluate a health outcome; hence was not eligible for registration with PROSPERO.

We conducted literature search in databases MEDLINE/PubMed and Scopus with the search string: (Pemphigus vulgaris) AND (Kinase OR phosphatase OR “p38 mitogen‐activated kinase” OR p38MAPK OR “protein kinase C” OR PKC OR “cyclin‐dependent kinase 2” OR CDK2 OR “tyrosine phosphatase” OR “bruton tyrosine kinase” OR BTK OR “mitogen‐activated protein kinase‐activated protein kinase 2” OR MK2 OR “extracellular signal‐regulated kinase” OR ERK OR Src OR “c‐Jun N‐terminal kinase” OR JNK OR “tyrosine kinase” OR TK OR “focal adhesion kinase” OR “FAK” OR “Phosphatidylinositol 3‐kinase” OR “PI3K”).

### 2.2 Selection criteria

Our inclusion criteria were in vitro, in vivo and human studies that evaluated the role of kinases in PV. Studies were eligible if a) they included PV patients or used PV‐IgG, PV serum, purified PV‐IgG to induce PV‐like phenotype, acantholysis, or intercellular detachment, and b) if these features could be inhibited or prevented by using specific molecule inhibitors, knock out of specific gene, silencing or inactivation of kinases. Non‐English articles, reviews, books, letters, and information presented at conferences were excluded. There was no publication year restriction. Full search details are provided in Figure 1.

**Figure 2:**
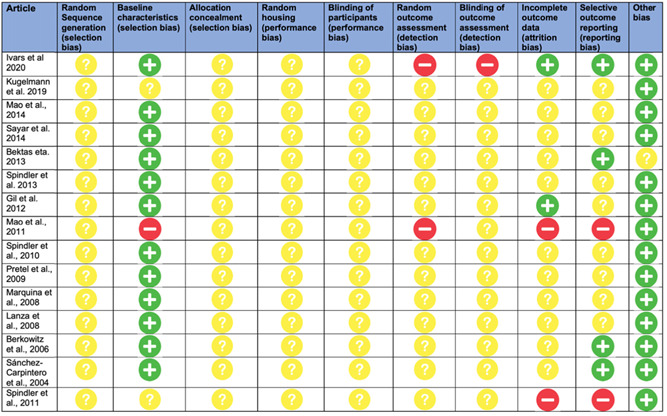
A risk of bias assessment questionnaire performed on in vivo studies utilizing SYRCLE's risk of bias tool for animal studies.

### 2.3 Data collection and quality assessment

In the initial phase, two reviewers were assigned for each database performing the search process and title screening. Non‐English articles were filtered out, manual screening was performed to exclude reviews, books, letters and information presented at scientific conferences. Duplicated articles were removed. In the second phase, titles and abstracts were screened independently by two reviewers to their relevance to “PV.” Disagreements were consulted by the senior author. In the final phase, articles were read in full text and screened in accordance with the inclusion criteria. A total of 35 were included in this systematic review.

A pilot testing of inter‐reviewer agreement was performed for each database. Kappa score demonstrates strong agreement between reviewers of PubMed (0.88), substantial agreement between reviewers of Scopus (0.76).

### 2.4 Risk of bias assessment

Assessment of risk of bias in included animal studies has been performed in this review using SYRCLE's risk of bias tool (Hooijmans et al., 2014). Risk of bias is independently reviewed based on 10 domains: sequence generation (selection bias), baseline characteristics (selection bias), allocation concealment (selection bias), random housing (performance bias), blinding (performance bias), random outcome assessment (detection bias), blinding (detection bias), incomplete outcome data (attrition bias), selective outcome reporting (reporting bias), and other sources of bias (other; Hooijmans et al., 2014). For each of these components, reviewers categorized the included animal studies regarding the risk of bias as either high, low, or unclear based on guidance in Hooijmans et al. (2014).

## 3 RESULTS

From a total of 2114 hits (183 articles from PubMed and 1931 from Scopus), 35 studies were included in the qualitative synthesis (Figure 1) that demonstrated pathogenicity of kinase activation in PV (Table 1). The articles were stratified based on the model implemented in the study, including PV sera (*n* = 13) and PV‐IgG (*n* = 22). All studies used in vitro, in vivo, or ex vivo models, a combination thereof, or human subjects.

**Table 1:** Pathogenic signaling mechanisms in PV—kinases and phosphatases

	Author	Year	Study type	Pathogenic agent	Target molecule	Kinase inhibitor
**1**	Berkowitz et al.	2006	In vivo	PV Sera	p38MAPK	**p38MAPK (SB202190 and SB203580)**
**2**	Murrell et al.	2021	Human	PV Sera	BTK	BTK and **Rilzabrutinib (oral BTK inhibitor)**
**3**	Cirillo et al.	2010	In vitro	PV Sera	PKC	PKC (Go6972)
**4**	Kowalewski et al.	1994	In vitro	PV Sera	PKC	**Protein kinase (H7)**
**5**	Berkowitz et al.	2005	In vitro	PV Sera	p38MAPK	**p38MAPK (SB202190)**
**6**	Frusić‐Zlotkin et al.	2006	In vitro	PV Sera	EGFR, ERK, and c‐Jun	**EGFR (AG1478) and ERK (PD98059)**
**7**	Cirillo et al.	2008	In vitro	PV Sera	PKC, p38MAPK, and CDK2	**Staurosporine (STS)**
**8**	Cirillo et al.	2014	In vitro	PV Sera	Src	**Src I‐1**
**9**	Sayar et al.	2014	In vivo	PV Sera	EGFR	EGFR (Erlotinib) and **Dual EGFR/ErbB2 (Lapatinib)**
**10**	Gil et al.	2012	In vivo	PV Sera	FAK (Y397/925)	**FAK**
**11**	Lanza et al.	2008	In vivo and in vitro	PV‐Sera	CDK2	Cdk2 (Roscovitine)
**12**	Mao et al.	2014	In vivo, in vitro, and human	PV Sera	MK2	**MAPKAP kinase 2 inhibitor (MK2I)**
**13**	Burmester et al.	2020	In vitro and human	PV Sera	ERK, MAP, Src, and Kinase C	**PKC and p38MAPK**
**14**	Saito et al.	2012	In vitro	PV IgG	p38MAPK and TK	**p38MAPK (SB202190) and Tyrosine kinase (Genistein)**
**15**	Kugelmann et al.	2019	In vitro	PV‐IgG	EGFR, ERK, and Src	**Src (PP2)**
**16**	Walter et al.	2019	In vitro	PV‐IgG	EGFR, Src, and ERK	**EGFR and Src**
**17**	Radeva et al.	2019	In vitro	PV‐IgG	MEK and ERK	**MEK (U0126)**
**18**	Walter et al.	2017	In vitro	PV‐IgG	p38MAPK, PKC, Src, and ERK	**p38MAPK (SB202190), PKC (Bim‐X)**, Erk (U0126), and **Src (PP2)**
**19**	Vielmuth et al.	2018	In vitro	PV‐IgG	p38MAPK and Src	**p38MAPK** (**SB202190 and SB203580)**
**20**	Lee et al.	2009	In vitro and in vivo	PV‐IgG	p38MAPK	p38MAPK (SB202190)
**21**	Delva et al.	2008	In vitro	PV‐IgG	TK	Tyrosine kinase (Genistein)
**22**	Rotzer et al.	2015	In vitro	PV‐IgG	c‐Scr and p38MAPK	**p38MAPK (SB202190) and c‐Scr (PP2)**
**23**	Chernyavsky et al.	2007	In vitro	PV‐IgG	Src and p38MAPK	**p38MAPK (PD169316) and Src (PP2)**
**24**	Vielmuth et al.	2015	In vitro	PV‐IgG	p38MAPK	**p38MAPK (SB202190)**
**25**	Ivars et al.	2020	In vivo	PV‐IgG	ADAM10, EGFR, Src, and p38MAPK	**Src (PP1), ADAM10, and EGFR**
**26**	Marquina et al.	2008	In vivo	PV‐IgG	TK	TK (Genistein)
**27**	Sánchez‐Carpintero et al.	2004	In vivo	PV‐IgG	TK, PLC, PKC, and Calmodulin	**TK (Genistein, Herbimycin). PLC (U‐73122). PKC (bisindolylmaleinamide), Calmodulin antagonist (W‐7 hydrochloride)**
**28**	Pretel et al.	2009	In vivo	PV‐IgG	Akt/mTOR, Src, P‐HER1, P‐HER2, P‐HER3, and EGF	mTOR (Rapamycin), Src (PP1), HER2, and HER3
**29**	Egu et al.	2020	Human	PV‐IgG	JAK3, STAT2, STAT4, and STAT6 (TYK2 kinases), and p38MAPK	**p38MAPK (KC706)**
**30**	Egu et al.	2019	Human	PV‐IgG	p38MAPK, Src, PKC, and ERK	**ERK**, PKC
**31**	Egu et al.	2017	Human	PV‐IgG	p38MAPK and AK23	**p38MAPK (SB202190)**
**32**	Mao et al.	2011	In vivo and in vitro	PV‐IgG	p38, p38MAPK siRNA, and p38a MAPK KO mice	**p38MAPK**
**33**	Spindler et al.	2010	In vivo and in vitro	PV‐IgG	cAMP, p38MAPK, and PKA	**PKA (H89)**
**34**	Bektas et al.	2013	In vivo and in vitro	PV‐IgG	EFGR and p38MAPK	**EGFR**(**AG1478) and p38MAPK (SB202190)**
**35**	Spindler et al.	2011	Ex vivo, in vitro, and human	PV‐IgG	PKC and Dsg3	**PKC**

*Note*: Mechanisms highlighted in bold demonstrate increased involvement regarding pathogenicity.

### 3.1 PV sera

A total of 13 studies used PV sera as their pathogenic agent. Six studies used in vitro models with HaCaT cells or human keratinocyte cells (Berkowitz et al., 2005; Cirillo et al., 2008, 2010, 2014; Frusić‐Zlotkin et al., 2006; Kowalewski et al., 1994). Three studies used in vivo models with neonatal mice (Berkowitz et al., 2006; Gil et al., 2012; Sayar et al., 2014). One study was a clinical trial in PV patients (human model; Murrell et al., 2021). One study used a combination of in vitro and in vivo models (Lanza et al., 2008), one study used a combination of in vitro, in vivo, and human models (Mao et al., 2014) and one study used in vitro and ex vivo models (Burmester et al., 2020). Inhibitors were from the CMGC, AGC, TK, and CAMK protein kinase groups. All 13 studies administered inhibitors before PV sera. Overall, the use of kinase inhibitors prevented or reduced acantholysis, keratinocyte dissociation, and blister formation.

### 3.2 PV‐IgG

A total of 22 studies used PV‐IgG as their pathogenic agent. Ten studies used in vitro models with HaCaT cells or human keratinocyte cells (Chernyavsky et al., 2007; Delva et al., 2008; Kugelmann et al., 2019; Radeva et al., 2019; Rötzer et al., 2015; Saito et al., 2012; Vielmuth et al., 2015, 2018; Walter et al., 2017, 2019). Four studies used in vivo models with neonatal mice (Ivars et al., 2020; Marquina et al., 2008; Pretel et al., 2009; Sánchez‐Carpintero et al., 2004). Three studies used models where PV‐IgG was administered to ex vivo human skin cultures (Egu et al., 2017, 2019, 2020). Four studies used a combination of in vitro and in vivo models (Bektas et al., 2013; Mao et al., 2011; Spindler et al., 2010) and one study used a combination of in vitro and ex vivo models (Spindler et al., 2011). Key inhibitors administered were from CMGC, AGC, and TK protein kinase groups. Pretreatment with inhibitors was conducted in 11 studies (Bektas et al., 2013; Chernyavsky et al., 2007; Delva et al., 2008; Egu et al., 2017, 2019; Ivars et al., 2020; Marquina et al., 2008; Rötzer et al., 2015; Saito et al., 2012; Sánchez‐Carpintero et al., 2004; Walter et al., 2017). Five studies administered PV‐IgG injection before inhibitor treatment (Mao et al., 2011; Radeva et al., 2019; Spindler et al., 2011; Vielmuth et al., 2018; Walter et al., 2019). Three studies delivered PV‐IgG injection and inhibitors at the same time (Egu et al., 2020; Kugelmann et al., 2019; Spindler et al., 2010). One study delivered inhibitors before and after PV‐IgG injection (Lee et al., 2009). PV‐IgG injection timing was not disclosed in one study (Vielmuth et al., 2015). Kinase inhibition was associated with inhibition of phosphorylation events associated with pemphigus acantholysis, prevented at least in part blister formation and PV‐IgG‐induced acantholysis.

### 3.3 Risk of bias assessment

A risk of bias assessment questionnaire was performed on in vivo studies utilizing SYRCLE's risk of bias tool for animal studies (Figure 2). A vast majority of parameters were not reported within articles, thus resulting in the studies attaining an unclear risk of bias with consequent dubious level of evidence. The baseline characteristics domain was well reported and established among most studies, whereby allocation concealment, random sequence generation, random housing and blinding of outcome assessment were not reported in a majority of studies.

## 4 DISCUSSION

**Figure 3:**
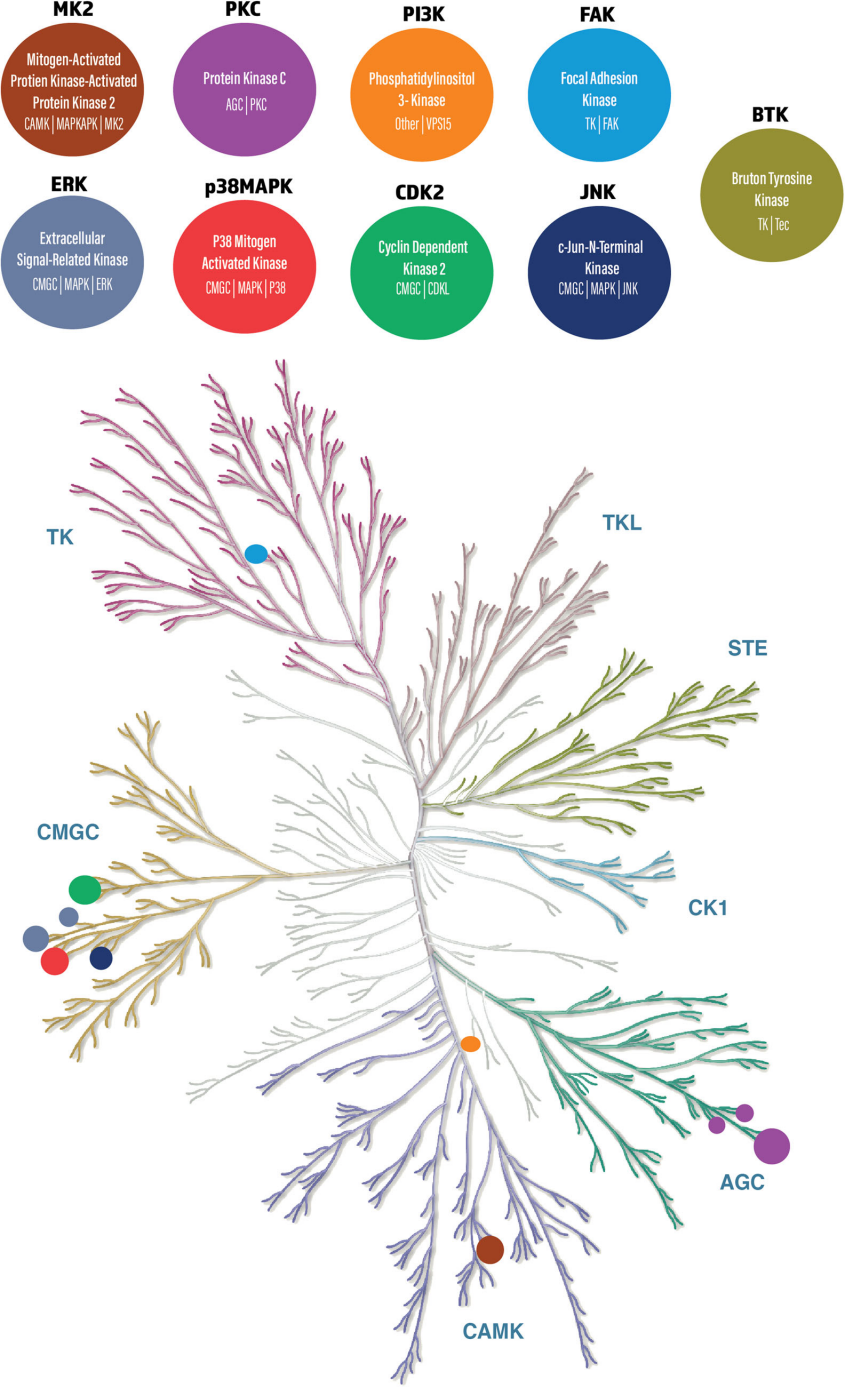
An illustration of the human kinome which comprises 538 kinases responsible for catalyzing protein phosphorylation, courtesy of Cell Signaling Technology, Inc. Kinases included in this review are highlighted in their respective categories.

Kinases are enzymes that regulate the biological activity, expression or localization of proteins via phosphorylation. They induce an active form of an inactive protein by means of a conformational change (Avendaño & Menendez, 2015). Kinases have been identified to contribute to the pathogenesis of PV via binding of autoantibodies in PV. This binding promotes the phosphorylation of kinases, which are hypothesized to induce PV‐associated acantholysis. Inhibition of such pathways in in‐vivo and in‐vitro models have been shown to reduce acantholysis in PV. Furthermore, BTK inhibitors have recently been tested in patients, with more research needed to understand their importance in preventing blistering. Overall, kinases that are pathogenically associated with PV acantholysis include several kinome branches such as CMGC (p38MAPK, cdk2, c‐JNK, ERK, and ASK1), ACG (PKC isoforms), CAMK (MK2 and PI3K), and TK (BTK, FAK, Src, and EGFR; Figure 3).

### 4.1 CMGC protein kinase group

#### 4.1.1 p38 mitogen‐activated protein kinases

The p38 mitogen‐activated protein kinases are a class of kinases belonging to the CMGC protein kinase group; these kinases are activated by a number of cellular stresses including growth factors, inflammatory cytokines, osmotic shock, and ultraviolet light (Mavropoulos et al., 2013). The p38 MAPK signaling pathway has been implicated in the regulation of cellular and humoral autoimmune responses (Mavropoulos et al., 2013), its main role is coordination of cytokine gene expression including tumor necrosis factor and interferon‐ϒ via transcriptional and posttranscriptional mechanisms (Mavropoulos et al., 2013). The signaling pathway of p38MAPK is known to play an important role in chronic inflammatory skin conditions (Mavropoulos et al., 2013). p38MAPK inhibitors are able to reduce skin inflammation in various mouse models of human skin conditions (Mavropoulos et al., 2013). Rapid and dose‐dependent phosphorylation of p38MAPK occurs after PV antibodies bind to cultured keratinocytes (Berkowitz et al., 2006). Inhibitors of p38MAPK have been found to prevent early cytoskeletal changes that are known to be associated with cell–cell adhesion loss, in human keratinocyte cultures (Berkowitz et al., 2006; Walter et al., 2017). In in vitro studies, selective inhibition of p38MAPK has been found to inhibit PV IgG‐dependent cell shrinkage and keratin aggregation (Chernyavsky et al., 2007). Inhibition of p38MAPK has been reported to prevent PV blistering in vivo (Berkowitz et al., 2006), demonstrating a potential therapeutic intervention for the disease (Mavropoulos et al., 2013).

#### 4.1.2 Cyclin‐dependent kinase 2

In addition to MAPKs, cyclin‐dependent kinases (CDKs) are of the most well studied and largest CMGC groups (Varjosalo et al., 2013). Beyond their well‐known role in regulating the cell cycle, CDKs also control human tumor suppressors activity (Varjosalo et al., 2013). For activation, CDK requires phosphorylation of the CDK subunit to fully activate the Ser/Thr protein kinase activity (Russo et al., 1996). In the neonatal mouse model of PV, it has been found that CDK2 inhibition through roscovitine can prevent acantholysis (Lanza et al., 2008). In addition, increased CDK2 expression in perilesional sites at suprabasal layers indicates CDK2 activation as the predecessor to blister formation (Lanza et al., 2008).

#### 4.1.3 c‐Jun N‐terminal Kinase

JNK is among a number of pathways that are triggered by PV‐IgG; anti‐Dsg3 antibodies in PV autoimmunity are for the most part related to signaling events regarding both JNK and p38MAPK (Marchenko et al., 2010). JNK is activated by environmental stresses and cytokines, JNK is also found to regulate the signaling pathway of cellular apoptosis, proliferation, and tissue morphogenesis (Ip & Davis, 1998). JNK is activated by dual phosphorylation of threonine and tyrosine residues carried out by MAP kinase kinases, MKK4 and MKK7 (Ip & Davis, 1998). JNK activation in response to cytokines and environmental stress suggests the potential contribution of JNKs signaling pathway to inflammatory responses (Ip & Davis, 1998). YAP is a protein known to act as transcriptional regulator, the regulation of YAP can be mediated by a number of kinases including JNK, p38MAPK, PKC, and SRC (Huang et al., 2021). Inhibition of JNK by SP600125 can cause YAP dysregulation (Huang et al., 2021); YAP dysregulation is detected in PV, this is not at all surprising given the implications of the previously mentioned kinases in PV pathogenesis (Huang et al., 2021). Dysregulation of YAP is implicated in pathogenesis of PV and implies the therapeutic potential of antioxidants in PV treatment (Huang et al., 2021).

#### 4.1.4 Extracellular‐signal‐regulated kinases

ERK is part of MAP kinase signaling cascades and is vital in a number of cellular processes including cell differentiation, migration, adhesion, proliferation, and cell survival (Pouysségur & Lenormand, 2016). ERK can be activated in a number of ways including via G protein‐coupled receptors stimulation and release of Gβγ subunits, activation of growth factor‐stimulated tyrosine kinase receptors, transactivation of growth factor tyrosine kinase receptors such as EGFR via tyrosine phosphorylation of receptor or proteolytic cleavage of membrane‐bound ligand (Roberts, 2012). Inhibition of the ERK pathway has been shown to prevent cell cohesion loss in cell cultures (Egu et al., 2019). ERK inhibition via U0126 inhibitor terminated PV‐IgG and AK23 autoantibody induced loss of cell adhesion in ST18 transfected monolayers (Radeva et al., 2019); in both HaCaT and NHEK cell lines, release of pro‐inflammatory molecules such as IL‐1*α*, IL‐6, TNF‐*α*, and IFN‐*γ* was found to not always be required for loss of cell cohesion induced by PV‐IgG (Radeva et al., 2019). Inhibition of ERK with U0126 has been found to reduce blister formation as well as prevent the decrease of desmosomes; in the same paper, the inhibition of PKC was not found to prevent suprabasal blister formation (Egu et al., 2019).

### 4.2 AGC protein kinase group

#### 4.2.1 Protein kinase C

PKC regulates signaling pathways that promote lipid hydrolysis (Newton, 1995), and many receptor pathways that intertwine with lipid pathways commonly result with activation of PKC via production of its second messenger (Newton, 1995). PKC is activated via binding of ligands or substrates (Newton, 1995). Inhibition of PKC using Bim‐X was found to block cell cohesion loss induced by PV‐IgG and AK23 (Walter et al., 2017). Staurosporin (STS), a protein kinase inhibitor, was found to decrease 3 PV‐specific phosphorylation events in keratinocytes, and found to prevent cell–cell detachment and acantholysis (Cirillo et al., 2008). Protein kinase inhibitor H7 was found to prevent acantholysis in a cell culture model system with PV serum (Kowalewski et al., 1994). In a human study conducted by Spindler et al. (2011), PKC inhibition was found to blunt the loss of keratinocytes and their adhesion in the presence of PV‐IgG‐induced Dsg3 depletion (Spindler et al., 2011); in the same study in an ex vivo model of human skin, PKC inhibition displayed prevention of Dsg3 depletion while in a mouse model PKC inhibition blocked blister formation. In another study, it was found that PKC was required for the expression of Dsg1 and Dsg3 to a lesser degree (Denning et al., 1998); the downregulation of PKC decreased the accumulation of Dsg1, Dsg3, and mRNA suggesting a transcriptional regulation effect (Denning et al., 1998). The findings of Osada et al. (1997) suggest a unique activation profile of PKC isomers and their possible role in intracellular event signaling mediation induced by PV‐IgG binding to Dsg3 in cultured human keratinocytes (Osada et al., 1997). 1995 Unlike PKC, PKA activation is also part of a keratinocyte rescue pathway in response to PV‐IgG and hence PKA inhibition would be detrimental. In one study, PKA signaling was found to in part mediate cAMP effect on keratinocyte recovery in an in vitro study in which PV‐IgG elevated cAMP levels, cAMP signaling was implicated in preventing cell adhesion loss by interfering with PV‐IgG‐induced p38MAPK activation (Spindler et al., 2010). In the same study, an in vivo model demonstrated an iso‐mediated cAMP increase which blocked blister formation induced by IgG‐PV patients completely (Spindler et al., 2010).

### 4.3 TK protein kinase group

#### 4.3.1 Tyrosine kinases

Receptor tyrosine kinases are important for the regulation of functions in healthy cells and have a critical role in oncogenesis (Gschwind et al., 2004). A study by Saito et al. (2012) found that using a tyrosine kinase inhibitor (SB202190) resulted in blocking of acantholysis, clustering, and endocytosis of Dsg3 in human skin caused by PV‐IgG. Moreover, Frusić‐Zlotkin et al. (2006) reported similar findings that a specific tyrosine kinase inhibitor (AG1478) blocked the apoptosis induction caused by PV‐IgG and overturned cell death, FasL appearance, and acantholysis.

#### 4.3.2 Bruton tyrosine kinase

BTK is an essential part of B cell receptor signaling in that it regulates the proliferation and survival of B cells. BTK is connected in cytokine receptor signaling and is known to bring together signaling proteins such as those in innate immunity as well as adaptive immunity (e.g., implicated in the toll‐like receptor pathway). BTK is critical in transcription regulation by inducing NF‐kappa‐B responsible for regulating expression of many genes (Yu et al., 2009). While not shown to bind directly to DNA, it is required to form functional DNA‐binding complexes (ARID3A) and has a dual role in the regulation of apoptosis (Uckun, 1998). In a study by Murrell et al. (2021) which utilized a BTK inhibitor, patients presented with absence of new lesions and healing of existing lesions within 4 weeks of commencing rilzabrutinib, a BTK inhibitor, with minimal to no prednisone equivalent corticosteroid (<0.5 mg/kg; Murrell et al., 2021). BTK inhibition may be, therefore, a promising treatment strategy in the management of PV. However, a recent Phase III PEGASUS trial that evaluated rilzabrutinib for the treatment of pemphigus did not meet its primary or key secondary endpoints (Sanofi, 2021).

#### 4.3.3 Focal adhesion kinases

FAK plays an essential role in cell migration, adhesion, disassembly of focal adhesions, cell cycle progression, cell proliferation and apoptosis, to name a few (Tilghman & Parsons, 2008). It forms multiunit signaling complexes with Src with activation which causes phosphorylation of tyrosine residues and creation of binding sites for substrates, effectors, and scaffold proteins (Schaller et al., 1999). Moreover, FAK regulates numerous signaling pathways such as the activation of PI3 and AKT1 signaling cascade (Reif et al., 2003). It further promotes the activation of MAPK1/ERK2, MAPK3/ERK1, and the map kinase signaling cascade (Hastings et al., 2019). A study by España et al. (2013) observed that FAK inhibitors could prevent PV acantholysis. They found that mice injected with PV‐IgG had an increased expression of nNOS in basal cells. nNOS has been found to contribute to acantholysis in PV by upregulation of phosphorylated FAK. Inhibition of nNOS was found to abolish clinical and histological findings of PV. Furthermore, Gil et al. (2012) reported similar findings of decreased suprabasal acantholysis induced by PV‐IgG with FAK inhibition, whereas infection of mice with PV‐IgG increased levels of FAK phosphorylation on tyrosine residues. Finally, Penneys (1996) reported no detection of FAK in normal keratinocytes as opposite to keratinocytes of PV, which were positive for FAK staining in acantholytic cells.

### 4.4 CAMK protein kinase group

#### 4.4.1 Mitogen‐activated protein kinase‐activated protein kinase 2

MK2 or MAPKAPK2 is involved in endocytosis, the production of cytokines, cell migration, cell cycle control, chromatin remodeling, DNA damage response, transcriptional regulation and the cytoskeleton reorganization. MK2 is phosphorylated via stress and activated by p38 alpha which regulates inflammatory cytokine production (e.g., TNF‐alpha; Beamer & Corrêa, 2021). A study by Mao et al. (2014) assessed the inhibition of MK2 which resulted in a loss of cell‐surface Dsg3, gross blisters and acantholysis. In humans with PV skin blisters, MK2 was activated and translocation of MK2 from the nucleus to the cytosol was further observed. Mao et al. (2014) also found that MK2 is activated in response to pathogenic anti‐Dsg1/3 PV monoclonal antibodies. Lastly, with silencing and inhibition of MK2 there was blocking of PV monoclonal antibody‐induced Dsg3 endocytosis and spontaneous blisters by PV monoclonal antibodies but in the neonatal mouse model, induced blisters were not prevented.

#### 4.4.2 Phosphatidylinositol 3‐kinase

The PI3K pathway regulates various cell processes including cytoskeleton rearrangement, proliferation, apoptosis and growth (Vivanco & Sawyers, 2002). A study by Burmester et al. (2020) was successful in identifying topical inhibition of signaling kinases as a novel therapeutic agent for PV. Specifically, they identified four molecules in PV IgG‐induced skin pathology including PI3K, MEK1, TrkA, and VEGFR2. A study by Lai et al. (2021) further found that PI3K is activated by certain stimuli or inflammation and that in active form PI3K phosphorylates AKT resulting in AKT/mTOR pathway activation (Lai et al., 2021).

### 4.5 Conflicting data

Egu et al. (2020) found inhibition of p38MAPK was not effective in preventing mucosal acantholysis. According to the study, both AK23 and mucosal‐dominant PV‐IgG induce blisters and associated ultrastructural changes in labial mucosa via a mechanism not dependent on p38MAPK signaling (Egu et al., 2020). Furthermore, a study from the same group found that inhibition of PKC was not effective to prevent suprabasal blister formation or ultrastructural alterations of desmosomes (Egu et al., 2019).

According to Lee et al. (2009), data showing treatment before IgG indicates inhibition of cytokeratin retraction (blocking of acantholysis); treatment after IgG selectively/fails to block cytokeratin retraction or acantholysis. Findings from Sayar et al. (2014) show Lapatinib significantly reduced blistering in the oral cavity, Erlotinib in epidermis; as suggested by EGFR gene deletion, none of the erlotinib (inhibiting EGFR) concentrations significantly reduced AK23‐induced blistering in the palate.

With regard to BTK, after the promising results of a Phase II trial that evaluated rilzabrutinib for the treatment of pemphigus, a recent press release (Sanofi, 2021) announced that the Phase III trial had not met its primary endpoints.

Finally, the results by Wei and Li (2021) were not included in our qualitative synthesis. The authors show that ASK1 inhibition prevents PV‐induced apoptosis rather than cell–cell detachment or acantholysis—whether or not these are the same mechanism is currently the subject of an intense debate. In mice, Trx2 overexpression reduced blister formation and was associated with repression of ask1 phosphorylation (Wei & Li, 2021). However, there was no direct evidence in this study that ask1 inhibition prevented blister formation.

## 5 LIMITATIONS

The current review considered in vitro models, in vivo models, human models, ex vivo, or a combination. However, due to the very limited number of clinical studies (Murrell et al., 2021), a risk of bias assessment was undertaken only for in vivo (animal) studies that met the inclusion criteria (Figure 2), and the results indicated that a significant amount of bias was likely. Furthermore, many of the studies did not mention the experimental conditions that form part of SYRCLE's assessment criteria; this resulted in several “unclear” responses leading to a limited bias assessment.

Observed inhibition of various target molecules resulted in improvements or no change of the phenotypic and histological characteristics of acantholysis associated with PV. However, a single kinase inhibitor and pathogenic agent concentration were used in most included studies. Therefore, a reliable dose–response effect has yet to be established for most kinase inhibitors.

Current literature focuses on in vivo mouse models and in vitro human keratinocytes to provide further insight into kinase involvement in PV. However, the expression of key molecules differs between humans and mice. These differences may result in the involvement of other mechanisms affecting cell cohesion and signaling in PV, and hence present a major challenge when attempting to translate these research findings to humans.

Furthermore, not all papers used language consistently when reporting kinase inhibitors, which possibly resulted in missed reporting of inhibitors, emphasizing the importance for class molecule data segregation. While there remains a potential to study targeted therapy modalities in the class of kinases, the feasibility of targeting these molecules in otherwise healthy individuals may be limited by the subsequent side effects associated with inhibition of such molecules in a healthy human.

Researchers performing this review examined the literature for PV for a specific class of intracellular molecules independently. Thus, the development of a comprehensive model to elucidate PV pathogenesis and cell signaling interactions amongst other class molecules involved in PV pathogenesis is beyond the scope of this review.

## 6 CONCLUSION

The aim of this review was to systematically evaluate classes of kinases in relation to pathogenicity in order to highlight molecules potentially suitable for targeted therapy in PV. By evaluating selected English papers from PubMed and Scopus which included in vitro, in vivo, and human studies and further extracting data and assessing risk of bias, the pathogenicity of kinases in PV was systematically assessed.

The kinase inhibitors that emerged as potential candidates for PV treatment those targetting p38MAPK, PKC, TK, c‐Src, EGFR, MEK/ERK, mTOR, BTK, and CDK. Kinase inhibition was found to reduce PV‐associated phosphorylation events as well as keratinocyte dissociation. Further, inhibition of kinases prevented acantholysis and blocked blister formation. The protocols and dosages used in experimental models are however an area for potential improvement as it can aid in predicting the prevention of blistering and acantholysis. Hence, future research may consider establishing a consistent reporting and dosing scheme to facilitate investigation into potential doses for human studies. In addition, it remains to be established how inhibition of target molecules would impact the physiology of humans across the health continuum.

In summary, this review has highlighted the complex molecular mechanisms of kinases that are related to the pathogenicity of PV. The kinase class of molecules discussed in this study display evidence supporting the potential for clinical translational research, establishing the requirement for more randomized controlled trials with human subjects in the future.

## CONFLICTS OF INTEREST

The authors declare no conflicts of interest.
